# Landouzy sepsis in a young adult with multiorgan failure: successful ECMO-assisted management using an individualized antituberculous regimen

**DOI:** 10.1186/s12879-026-13324-4

**Published:** 2026-04-15

**Authors:** Lukas van de Sand, Simon Dubler, Stefanie Bertram, Jana Luisa Aulenkamp, Nina Vanessa Pirschtat, Oliver Witzke, Yael Hegerfeldt, Markus Zettler

**Affiliations:** 1 Department of Infectious Diseases and Nephrology, University Hospital Essen, University Duisburg-Essen, Hufelandstraße 55, 45147 Essen, Germany; 2 Department of Anaesthesiology and Intensive Care Medicine, University Hospital Essen, University Duisburg- Essen, Essen, Germany; 3 Institute of Pathology, University Hospital Essen, University Duisburg-Essen, Essen, Germany; 4 Department of Pulmonary Medicine, University Hospital Essen- Ruhrlandklinik, University Duisburg-Essen, Essen, Germany

**Keywords:** Landouzy sepsis, ARDS, VV-ECMO, VVV-ECMO, Amikacin ototoxicity

## Abstract

**Background:**

Fulminant disseminated tuberculosis (TB) presenting as Landouzy sepsis is rare but carries high mortality. When complicated by acute respiratory distress syndrome (ARDS) and multiorgan failure, evidence for optimal management, including extracorporeal membrane oxygenation (ECMO) and modified antituberculous regimens, remains limited.

**Case presentation:**

We describe a 23-year-old man with miliary tuberculosis who developed Landouzy sepsis, ARDS, hepatic dysfunction, and refractory hypoxemia requiring veno-venous (VV) and subsequent veno-veno-venous (VVV) ECMO for six weeks. Standard anti-TB treatment for susceptible mycobacteria was withheld due to liver injury most likely related to antituberculous drug toxicity. Despite initial treatment with isoniazid, rifampicin, and ethambutol, rising bilirubin prompted rifampicin discontinuation and transition to an individualized regimen including ethambutol, isoniazid, levofloxacin, bedaquiline, and amikacin. Corticosteroid therapy was started for suspected secondary organizing pneumonia during persistent hypercapnic respiratory failure, followed by clinical improvement. The course was complicated by thrombocytopenia, pneumothorax, arrhythmia, plasma exchange–dependent hyperbilirubinemia, and amikacin-associated sensorineural hearing loss. Microbiological clearance was achieved, organ dysfunction progressively recovered, and first-line therapy was successfully reintroduced after stabilization. The patient was ultimately discharged without oxygen requirement and started a rehabilitation program.

**Conclusions:**

This case highlights that even life-threatening tuberculosis with multiorgan failure can be survivable. Early diagnosis, flexible adaptation of anti-TB therapy, and prolonged ECMO support within multidisciplinary care were key to clinical recovery.

## Background

Tuberculosis (TB) remains one of the leading infectious causes of morbidity and mortality worldwide, despite being treatable and preventable. According to the World Health Organization, approximately 10.8 million new TB cases occur annually, with the majority of cases arising in low- and middle-income countries [1]. Miliary tuberculosis is a rare disseminated form caused by hematogenous spread of *Mycobacterium tuberculosis*. It accounts for about 3% of all TB cases and 12% of extrapulmonary forms [2]. It is more common in immunocompromised patients, especially those with HIV infection, but can also occur in immunocompetent individuals. Miliary TB may involve several organs and can lead to severe systemic inflammation and multi-organ dysfunction [3].

In very rare cases, disseminated TB can cause septic shock, known as Landouzy sepsis. It represents a fulminant form of TB with high mortality, often exceeding 70% [4]. Landouzy sepsis is mainly reported in patients with advanced immunosuppression, but isolated cases in healthy young adults have been described [4]. When TB presents with acute respiratory distress syndrome (ARDS) and multi-organ failure, the prognosis is particularly poor. In selected cases, extracorporeal membrane oxygenation (ECMO) can serve as rescue therapy [5, 6]. Prompt diagnosis and early treatment are key elements in sepsis management. Delays in antimicrobial therapy increase the risk of progression to septic shock and death, both in general sepsis and in TB sepsis [7].

In this report, we describe the case of a 23-year-old man with Landouzy sepsis complicated by ARDS, who was successfully treated with VV/VVV-ECMO and an individualized antituberculous regimen [8]. We aim to raise awareness that tuberculosis can present as fulminant sepsis even in non-endemic settings and to show that adapted anti-TB therapy can be effective even in patients with multi-organ failure.

## Case presentation

### Initial presentation

A 23-year-old man of Guinean origin was admitted to a regional hospital with suspected sepsis. He reported a one-week history of progressive malaise, productive cough, dyspnoea, and generalized weakness. One week prior, he had been evaluated by his general practitioner and empirically treated with oral antibiotics for a presumed respiratory tract infection (upper or lower), without clinical improvement. On admission, he was febrile (39 °C) and tachycardic (heart rate up to 133 bpm) with respiratory distress requiring supplemental oxygen at 2 L/min. A chest X-ray revealed bilateral infiltrates consistent with an atypical pneumonia. A transthoracic echocardiogram performed at the referring hospital showed preserved left ventricular systolic function, no relevant valvular abnormalities, no pericardial effusion, and no evidence of right heart strain, making a primary cardiac cause of the bilateral infiltrates unlikely. Empiric antibiotic therapy with ampicillin/sulbactam and azithromycin was initiated, alongside intravenous fluid resuscitation. However, his respiratory condition deteriorated the following day, necessitating transfer to the intermediate care unit for non-invasive ventilation (NIV). Arterial blood gas analysis prior to NIV initiation under high-flow oxygen therapy (12 L/min oxygen with a total flow of 15 L/min) showed respiratory alkalosis (pH 7.48) with hyperventilation (PaCO₂ 29 mmHg) and insufficient oxygenation (PaO₂ 65 mmHg). NIV was initiated using a PSV mode (Elisa 500, Löwenstein Medical SE & Co. KG, Bad Ems, Germany) with an FiO₂ of 0.55, inspiratory positive airway pressure (IPAP) of 16 mbar, positive end-expiratory pressure (PEEP) of 8 mbar, and pressure support of 8 mbar. Despite this intervention, intubation and transfer to the intensive care unit (ICU) were required due to progressive respiratory failure and hemodynamic instability. Microbiological testing, including blood and respiratory cultures, remained negative. Chest computed tomography (CT) ruled out pulmonary embolism but revealed paraaortic lymphadenopathy as well as diffusely distributed, small patchy, partly confluent bilateral pulmonary consolidations. The patient had already developed severe ARDS, with a reported PaO₂/FiO₂ ratio < 100 mmHg, requiring invasive mechanical ventilation and prone positioning; however, refractory hypoxemia persisted and prone positioning did not result in relevant improvement. Consequently, the patient was transferred to the university hospital for initiation of veno-venous extracorporeal membrane oxygenation (VV-ECMO) therapy.

### Course at the university hospital

**Fig. 1:**
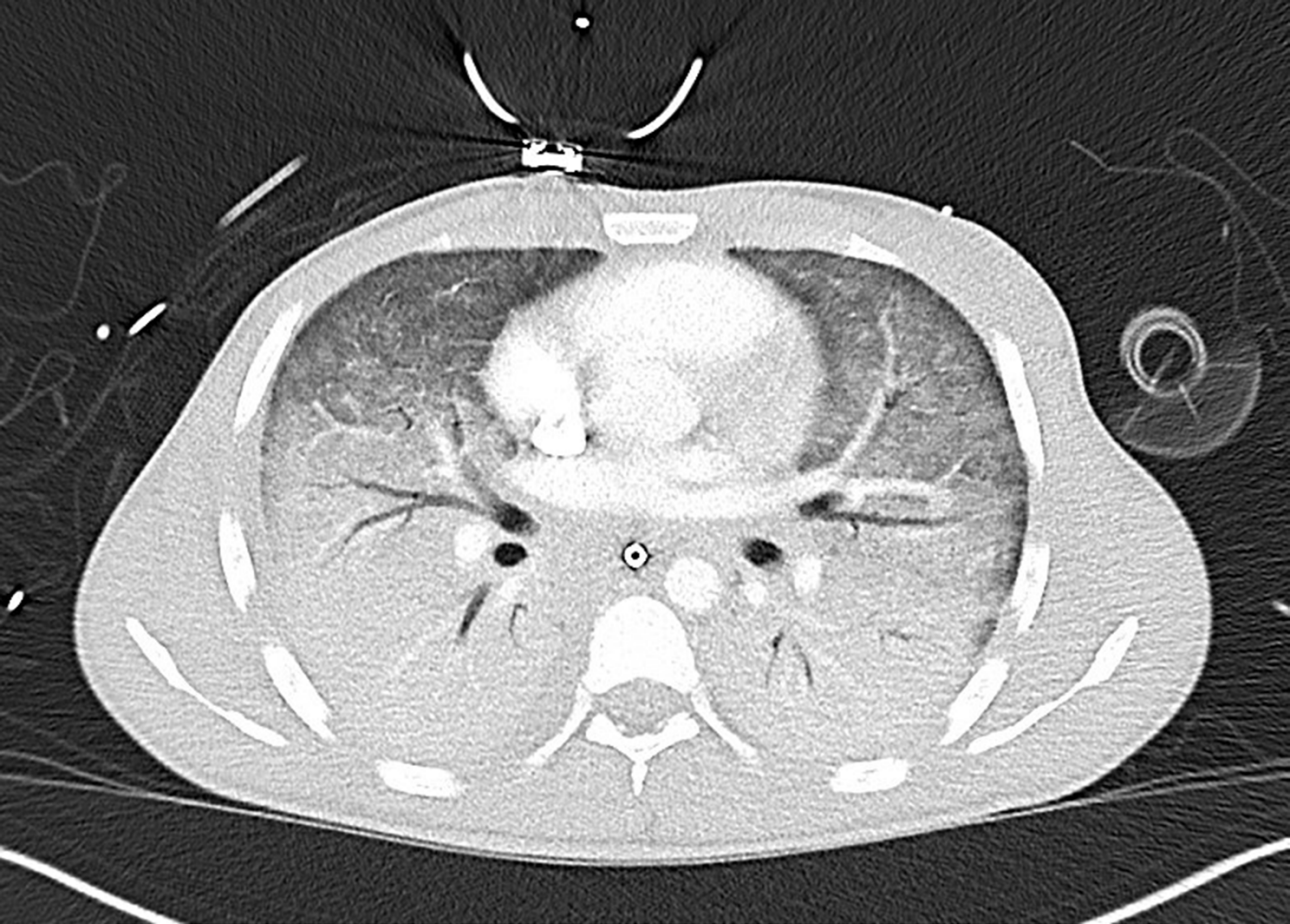
Chest CT at transfer to the university hospital. Progressive ARDS with increasing bilateral consolidations and ground-glass opacities, predominantly in the lower lobes. Likely reactive lymphadenopathy is present

On August 10th, 2025, VV-ECMO (Cardiohelp, Getinge, Rastatt, Germany) was initiated following uncomplicated bifemoral cannulation by the ECMO team. The patient was subsequently transported to our tertiary care ICU. Upon arrival, he was sedated, orotracheally intubated, and both hemodynamically and respiratory unstable, requiring norepinephrine at 0.2 µg/kg/min. Under pressure-controlled ventilation (FiO₂ 1.0, Pmax 25 mbar, PEEP 15 mbar) and VV-ECMO support (blood flow 3.0 L/min, sweep gas 1 L/min with 100% O₂), arterial blood gases revealed a PaO₂ of 82 mmHg, PaCO₂ of 53 mmHg, and pH 7.27. Upon arrival at our ICU, ECMO settings were adjusted to a blood flow of 3.2 L/min and a sweep gas flow of 2 L/min with an FiO_2_ of 1.0. Hemodynamic monitoring via a pulmonary artery catheter demonstrated a cardiac output of 5.4 L/min and a systemic vascular resistance of 581 dyn·s·cm⁻⁵ under the same norepinephrine dose. The pulmonary vascular resistance (4.86 Wood units) and mean pulmonary arterial pressure (29 mmHg) were elevated, consistent with pulmonary hypertension. Bronchoscopy revealed a small amount of thick, clear bronchial secretions and diffusely inflamed, contact-vulnerable mucosa. Extensive microbiological sampling, including bronchoalveolar lavage, was performed. Chest CT showed diffuse bilateral infiltrates, partly consolidating and partly ground-glass opacities (Fig. 1). Cranial CT demonstrated a small, atypical intracerebral hemorrhage, while abdominal CT revealed ascites and paraaortic lymphadenopathy.

Microbiological testing of sputum returned positive for *Mycobacterium tuberculosis* (PCR), and acid-fast bacilli ( + + positive) were confirmed on microscopy. The patient tested negative for HIV as well as for hepatitis B and C infection. As an interdisciplinary team, including infectious disease specialists, we considered these findings consistent with possible Landouzy sepsis due to possibly disseminated tuberculosis. Given hepatic dysfunction in the setting of multiorgan failure, the standard treatment was started in a modified regimen without pyrazinamide that is known for its high hepatotoxic potential. Acknowledging that rifampicin and isoniazid also carry well-described hepatotoxic risks, treatment was started with rifampicin (10 mg/kg), isoniazid (5 mg/kg), and ethambutol (15 mg/kg) under close monitoring of liver function parameters, as isoniazid and rifampicin are the most powerful agents in the treatment of TB.

**Fig. 2:**
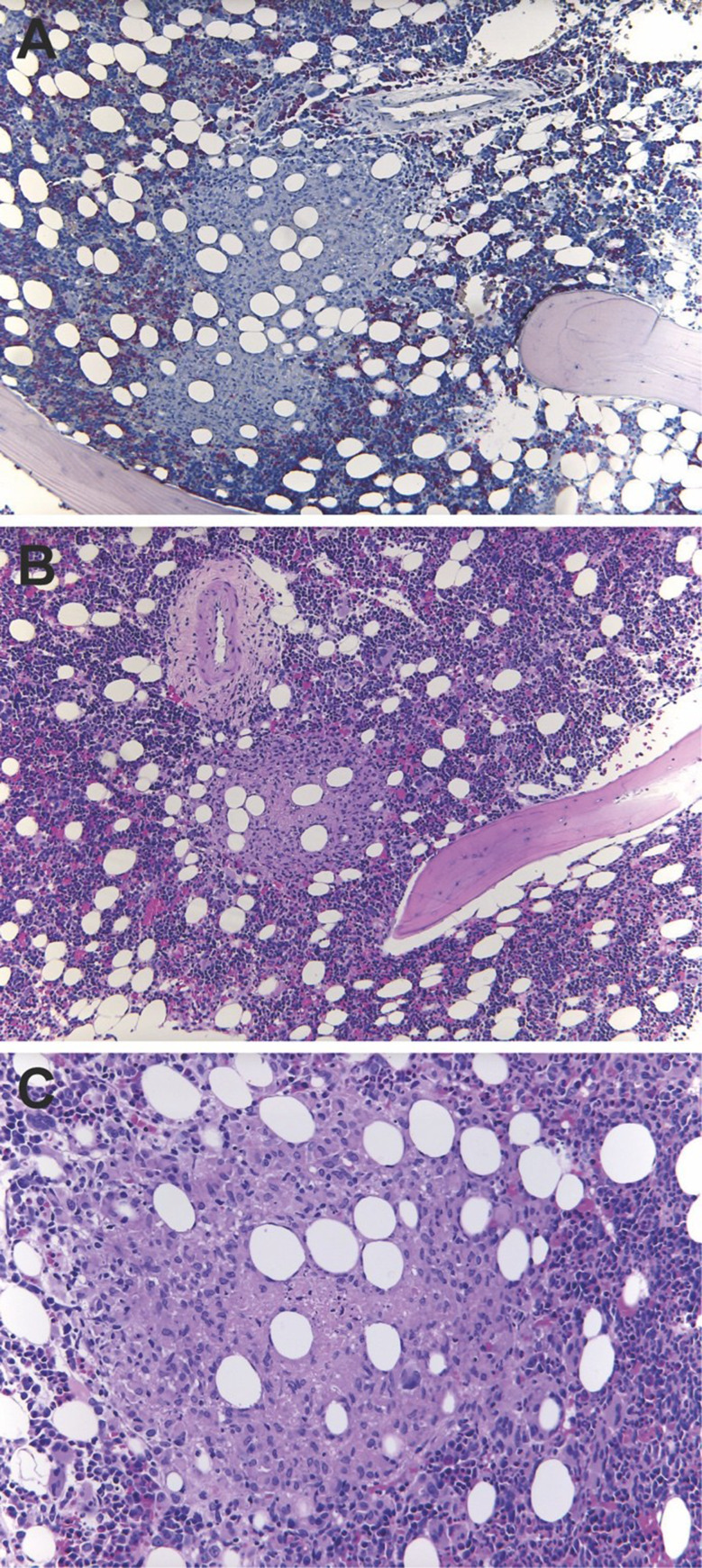
Bone marrow core biopsy (**A** Naphthol AS-D Chloroacetate Esterase Stain 100x, **B** H&E 100x, **C** H&E 200x). Normocellular hematopoietic marrow with numerous epithelioid cell granulomas showing central caseating necrosis

During the following days, the patient developed a marked increase in total bilirubin (up to 20.1 mg/dL), while transaminase levels remained only mildly elevated. In addition, progressive thrombocytopenia was noted. Because of unexplained cytopenia in combination with paraaortic lymphadenopathy and hepatomegaly, a bone marrow biopsy was performed for differential diagnostic evaluation. Histology revealed granulomatous inflammation (Fig. 2), and PCR testing of bone marrow tissue was positive for *M. tuberculosis*, confirming disseminated tuberculosis. However, blood samples tested negative for *M. tuberculosis*. Subsequent molecular resistance testing for *M. tuberculosis* returned negative for drug resistance.

**Fig. 3:**
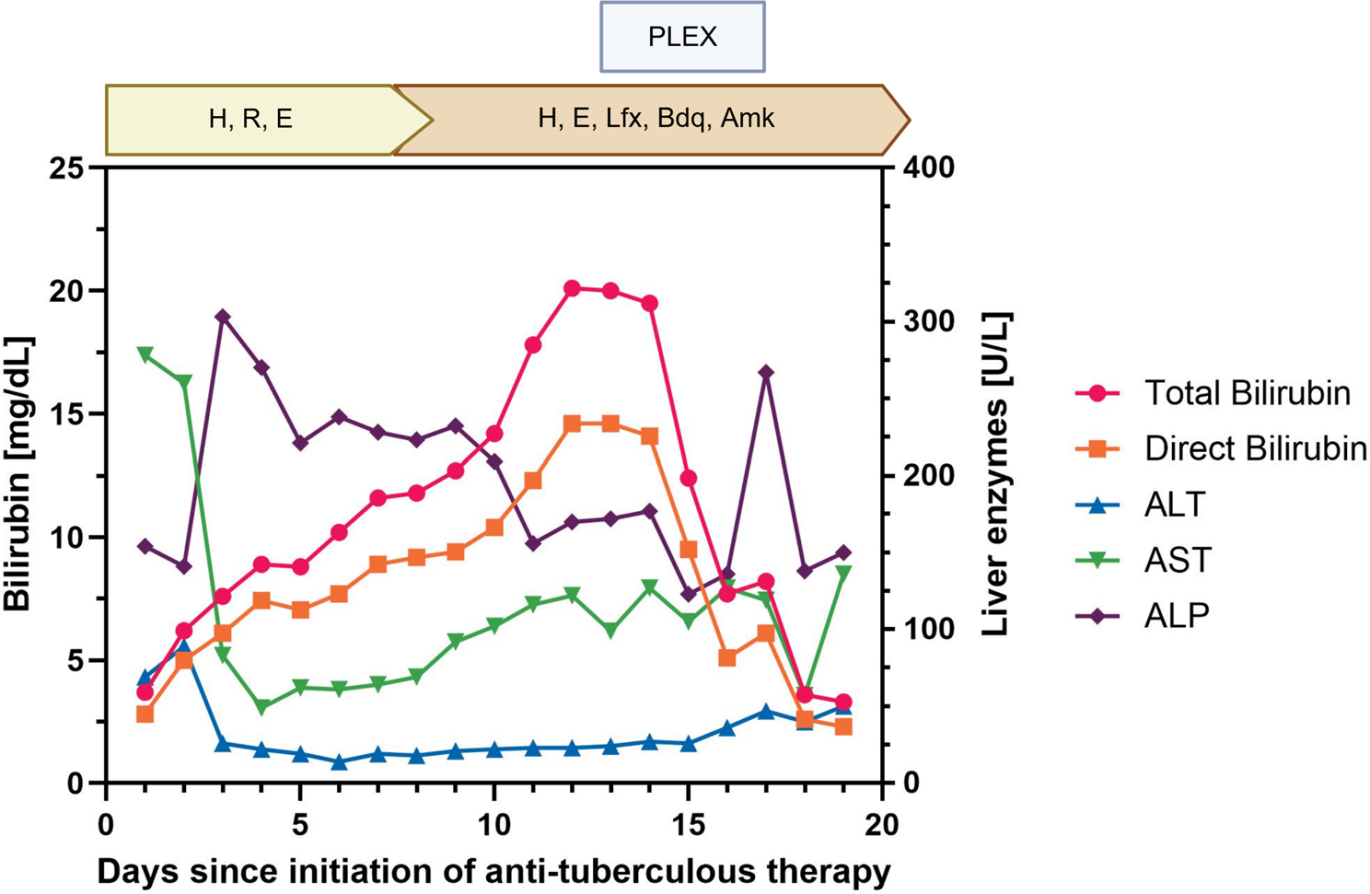
Time course of liver function parameters and treatment modifications during the clinical course. Total bilirubin (left y-axis) and liver enzymes (right y-axis) are shown in relation to days since initiation of anti-tuberculous therapy. Key clinical events, including changes in antituberculous regimens and plasma exchange therapy, are indicated along the time axis. Abbreviations: ALT, alanine aminotransferase; AST, aspartate aminotransferase; ALP, alkaline phosphatase; H, isoniazid; R, rifampicin; E, ethambutol; Lfx, levofloxacin; Bdq, bedaquiline; Amk, amikacin; PLEX, plasma exchange

After approximately one week of initial antituberculous therapy, the regimen was discontinued because of ongoing hepatic dysfunction, primarily driven by rapidly progressive hyperbilirubinemia, and replaced with an individualized treatment approach. The pattern of liver injury was characterized by markedly elevated bilirubin and alkaline phosphatase levels, while transaminases (ALT/AST) remained only mildly elevated, suggesting a predominantly cholestatic rather than hepatocellular injury pattern (Fig. 3). As previously described, isoniazid-related hepatotoxicity typically presents with significant transaminase elevation, whereas rifampicin is more commonly associated with cholestatic injury; therefore, rifampicin was considered the most likely causative agent and was discontinued [9, 10]. The R-factor at the time of rifampicin discontinuation was approximately 0.25, indicating a cholestatic pattern of liver injury. Isoniazid was continued due to its strong early bactericidal activity and the absence of biochemical evidence suggesting isoniazid-induced hepatocellular injury. We therefore initiated an individualized five-drug regimen, consisting of: Ethambutol (15 mg/kg), Isoniazid (5 mg/kg), Amikacin (15 mg/kg, maximum 1 g daily), Bedaquiline 400 mg daily for 14 days, followed by 200 mg three times per week and Levofloxacin 1000 mg daily. Given the patient’s critical condition and progressive multiorgan failure, this regimen deliberately deviated from national and international guidelines and was selected to maximize antimycobacterial efficacy while minimizing additional hepatotoxic risk. Therapeutic drug monitoring, which is not routinely recommended in standard tuberculosis treatment, was undertaken because of the patient’s critical illness, multiorgan failure, and the use of an individualized, non-guideline-based regimen. Drug levels of amikacin, isoniazid, ethambutol, and levofloxacin were measured using standard laboratory methods. Measured concentrations were largely within established therapeutic target ranges as reported in pharmacokinetic studies, with target peak concentrations of approximately 35–45 mg/L for amikacin, 3–6 mg/L for isoniazid, and 2–6 mg/L for ethambutol [11]. One amikacin level was markedly elevated at 75 mg/L, whereas subsequent measurements (51, 30, 47, and 34 mg/L) were within or close to the target range. Levofloxacin levels were within the expected range except for one measurement slightly below the target (6.7 mg/L; reference 8–13 mg/L). Concurrently, the patient experienced a rapid clinical deterioration characterized by progressive hepatic failure, increasing catecholamine requirements. Despite escalation of ECMO support (blood flow up to 3.5 L/min, sweep gas flow 4 L/min, FiO_2_ 1.0), persistent hypoxemia was observed. Therefore, an additional 17 Fr (23 cm) drainage cannula was inserted into the right internal jugular vein, resulting in a triple-site veno-veno-venous (VVV) ECMO configuration with bifemoral drainage and jugular return. Following this adjustment, ECMO blood flow was increased to 6.0–6.5 L/min and sweep gas flow to 8 L/min (FiO_2_ 1.0), resulting in improved systemic oxygenation.

The VVV-ECMO was maintained for four additional days and gradually de-escalated as pulmonary function slowly improved. Systemic anticoagulation was administered using unfractionated heparin, with a target activated partial thromboplastin time (aPTT) of 45–50 s, and was closely monitored and adjusted throughout the prolonged ECMO course. Decannulation of the third canula was performed uneventfully. Due to persistent hepatic dysfunction, extracorporeal plasma separation and adsorption were initially attempted but proved ineffective because of recurrent circuit clotting. The patient therefore subsequently underwent five sessions of therapeutic plasma exchange. The observed hyperbilirubinemia showed a more rapid decline following the initiation of plasma exchange therapy, suggesting a relevant contribution of this intervention to bilirubin clearance during the acute phase. The patient also received multiple platelet transfusions for therapy-refractory thrombocytopenia and repeated red blood cell transfusions due to ongoing anemia. Serial transthoracic echocardiographic assessments during the ICU stay did not demonstrate signs of right heart strain or acute cor pulmonale. From mid-September onward, the patient became increasingly awake and responsive. ECMO blood flow and sweep gas rates were gradually reduced in parallel with improved gas exchange. During the course of treatment, a right-sided pneumothorax developed and was managed by chest tube placement via mini-thoracotomy. The drain had to be maintained for several weeks, as repeated clamping attempts were unsuccessful. Later in the course, a contralateral pneumothorax occurred and was likewise treated successfully with chest tube placement via mini-thoracotomy. Additionally, a transient complete atrioventricular block (third-degree AV block) occurred, requiring temporary transvenous pacing, which was removed after a few days without further conduction abnormalities.

A 24-hour ECMO weaning trial at the end of September, was successful, allowing for complete VV-ECMO removal the following day. The patient experienced significant anxiety during the prolonged ICU course and received structured psychological support alongside targeted pharmacological measures. Enteral nutrition via nasogastric tube and early mobilization were consistently implemented as part of supportive care. Also wake up periods and spontaneous breathing trials were started early on ICU as part of the treatment strategy. After three consecutive negative sputum smears for acid-fast bacilli, the patient was deisolated and transferred to the pulmonary weaning unit at the beginning of October for further rehabilitation.

### Weaning, recovery and outcome

**Fig. 4:**
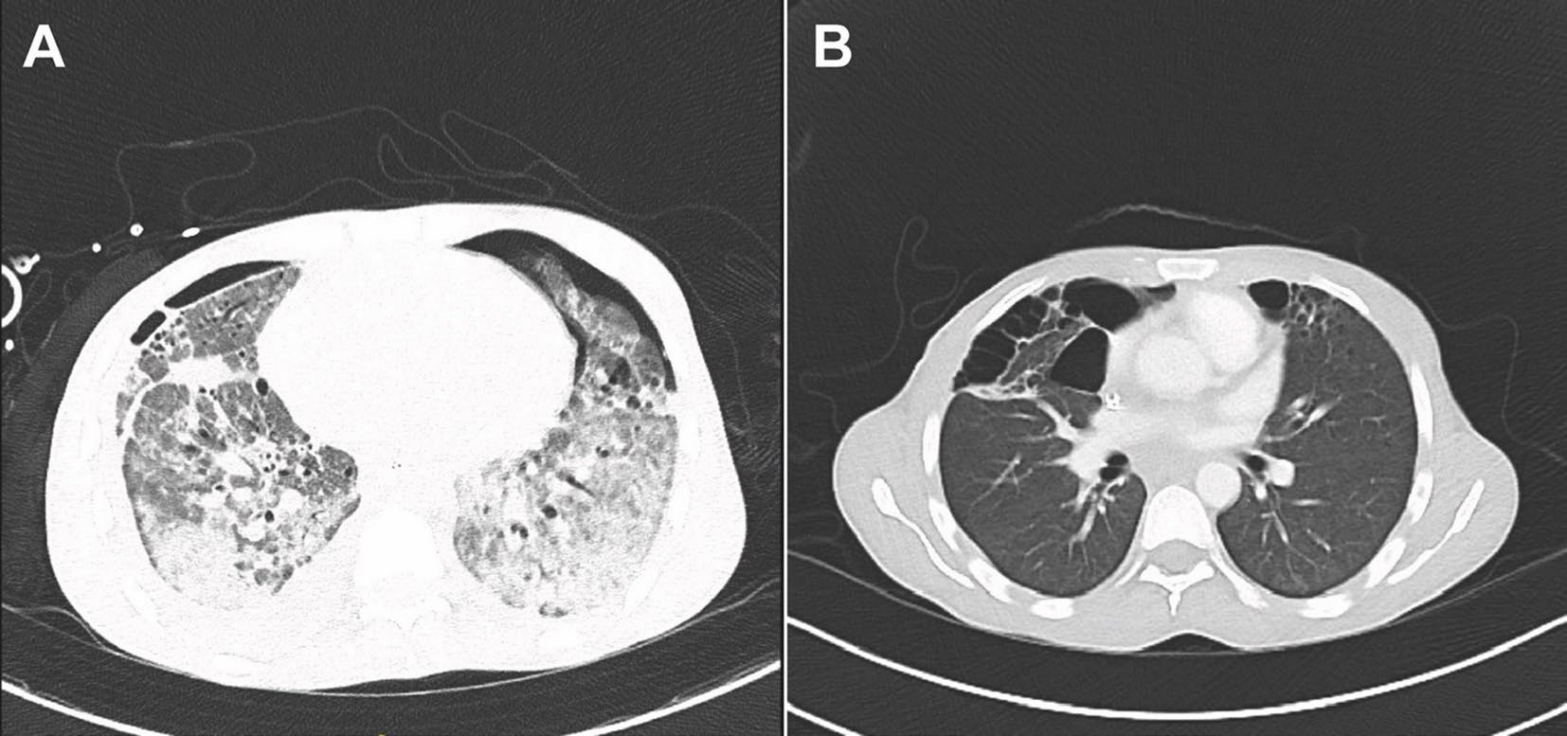
**A** Chest CT at beginning of spontaneous breathing trials. Progressive, patchy to confluent bilateral consolidations, most pronounced in the lower lobes, with underlying bronchiectasis in the setting of TB-related ARDS. Increasing ground-glass opacities in the upper lobes. New pleural effusions (right > left). On the right, air inclusions and adjacent pleural thickening are present. In addition, a new small ventral pneumothorax is present on the left side. **B** Follow-up chest CT on the medical ward. New, marked bullous changes with interval improvement of prior consolidations and ground-glass opacities

Initially, the patient required deep sedation to ensure adequate ventilation due to poor pulmonary compliance. Follow-up chest CT imaging revealed persistent ground-glass opacities and features suggestive of organizing pneumonia (Fig. 4A). Differential cytology of bronchoalveolar lavage showed only a mild lymphocytic predominance (15.3%). In the presence of cytomegalovirus detection, we initiated antiviral therapy with ganciclovir and started prednisolone at 50 mg (1 mg/kg) daily for suspected organizing pneumonia, alongside cotrimoxazole prophylaxis for *Pneumocystis jirovecii*. A comprehensive infectious work-up, including bronchoalveolar lavage and blood cultures with extended testing for fungal pathogens, revealed no evidence of concomitant fungal infection.

During the weaning process, the patient exhibited persistent hypercapnia, with pCO₂ values up to approximately 70 mmHg. Under continued anti-TB treatment, broad-spectrum antibiotic coverage with meropenem was administered as empiric therapy. Inflammatory parameters declined steadily, allowing discontinuation of meropenem in mid-October. Nevertheless, respiratory function improved, and from mid-October, spontaneous breathing trials via the pre-existing tracheostomy with speaking valve were initiated and progressively prolonged. Sedative medication was tapered gradually.

During this phase, clinical signs of sensorineural hearing loss became apparent, most likely attributable to amikacin therapy. By late October, the patient had regained sufficient respiratory and hemodynamic stability to be decannulated and transferred from the ICU to the infectious disease ward for continued rehabilitation. Follow-up chest CT on the ward showed marked improvement of pulmonary infiltrates. The scan also revealed new, marked bullous changes (Fig. 4B). Because the prior regimen was individualized and caused significant adverse effects, including likely amikacin-induced deafness, we adjusted therapy. Drug susceptibility testing showed a fully susceptible strain. We therefore continued treatment with isoniazid and rifampicin. Prednisolone was continued for one month after weaning and then gradually tapered.

## Discussion and conclusions

Severe infectious diseases leading to multiorgan failure and the need for prolonged intensive care are rare in young adults without prior comorbidity. Fulminant forms with a septic presentation are rare, reported in roughly 3% of TB cases [2]. Such patients challenge ICU teams with competing priorities. This case shows that survival is possible despite multiorgan failure. It underscores the value of interdisciplinary teamwork and early input from infectious disease specialists, sharpened diagnostics and therapy. Close coordination among critical care, infectious diseases, microbiology, radiology, pathology, and ECMO teams was decisive. Early suspicion, rapid diagnostics, and timely adaptation of antimycobacterial therapy were key to the outcome. In addition, dedicated nursing care, early and sustained physiotherapy with progressive mobilization, and psychosocial support played an important role during the prolonged ICU stay.

Our patient had miliary TB and survived 6 weeks of VV/VVV-ECMO, exceeding the duration usually reported as favourable [6]. A recent multicentre cohort study reported an overall survival rate of 51% after 90 days, with better outcomes for miliary TB than for cavitary TB under VV-ECMO (81% vs. 46%) [6]. The study concludes that ECMO may be justified in TB-related ARDS. Our patient mirrored complication patterns described in a systematic review of ECMO in TB [12]. Complications were common in that analysis, affecting roughly half of treated cases. Thrombocytopenia and pneumothorax were frequent, as in our case. Intracranial haemorrhage was reported in a minority of patients.

Due to the critical clinical condition of our patient and the presence of severe hepatic dysfunction, a highly effective antituberculous regimen with the lowest possible risk of additional hepatotoxicity was required. Standard care for drug-susceptible pulmonary TB now includes the 4-month 2HPMZ/2HPM treatment regimen as recommended by CDC and WHO; as an alternative, the traditional 6-month 2HRZE/4HR regimen remains in use (isoniazid [H], rifapentine [P], moxifloxacin [M], pyrazinamide [Z], rifampicin [R], and ethambutol [E]) [13]. Pyrazinamide is a well-recognized cause of clinically apparent, sometimes fatal, liver injury. It can also cause transient, asymptomatic enzyme rises. Given the patient’s hyperbilirubinemia, we did not start pyrazinamide [14]. In a larger cohort study, appropriate initial therapy including isoniazid and rifampicin, started early after shock onset, was associated with better survival than delayed or non-standard regimens [7]. Although pyrazinamide had been omitted from the regimen due to hepatotoxicity concerns, total bilirubin rose sharply from 3.7 to 20 mg/dL within a few days. Rifampicin has paradoxical effects on bilirubin. Total and indirect bilirubin often rise in the first days of therapy, then fall below baseline. Prominent increases in direct and total bilirubin can also occur within weeks, sometimes without overt liver injury. In combination regimens the risk of true hepatotoxicity increases, and injury from rifampicin typically begins within 1–6 weeks [15]. We therefore discontinued rifampicin and continued with an individualized regimen consisting of isoniazid, ethambutol, levofloxacin, amikacin and bedaquiline. Although isoniazid is also associated with hepatotoxicity, it was continued because of its well-established rapid bactericidal activity [16].

Contemporary evidence supports the potent activity of bedaquiline and later-generation fluoroquinolones as core agents for difficult or drug-resistant TB, and their inclusion in modern regimens has improved treatment success [17, 18]. Aminoglycosides such as amikacin retain strong bactericidal activity against *M. tuberculosis* and have been included historically in salvage and MDR regimens. However, systemic aminoglycosides carry a well-documented risk of irreversible hearing loss is a frequent and serious adverse outcome in TB programs that used injectables [19]. Close therapeutic drug monitoring (TDM) and audiometric surveillance are therefore essential when amikacin is used, but they cannot fully prevent the occurrence of adverse drug reactions. ECMO may significantly alter the pharmacokinetics of antimicrobials, due to increased volume of distribution, drug sequestration within the extracorporeal circuit, and changes in drug clearance in critically ill patients [20]. Consequently, TDM is particularly important in patients receiving ECMO to ensure adequate drug exposure while minimizing the risk of both underdosing and toxicity.

Although this specific combination of antituberculous agents has not been previously described, our patient demonstrated a favourable treatment response, evidenced by the clearance of respiratory samples (no detectable acid-fast bacilli on microscopy and subsequent de-isolation), successful reintroduction of rifampicin and isoniazid without recurrent toxicity, and clinical recovery allowing hospital discharge without supplemental oxygen.

Secondary organizing pneumonia likely contributed to delayed pulmonary recovery. Although corticosteroids are widely used to treat organizing pneumonia, high-quality evidence supporting their use is limited [21]. Recent evidence suggests a potential survival benefit of corticosteroid therapy in tuberculosis-associated ARDS [22], although the use of steroids remains controversial and should be considered on an individual basis. The temporal association between corticosteroid initiation and improved respiratory mechanics in our case is therefore intriguing, but it cannot definitively be attributed to steroids given the natural history of ARDS and concurrent therapies.

In conclusion, we want to highlight that early diagnosis and prompt initiation of therapy are crucial in severe TB. Even prolonged ECMO support can lead to favourable outcomes when paired with intensive multidisciplinary management, and flexibility in adapting antituberculous regimens may be both necessary and effective in complex clinical situations. However, it must be emphasized that the regimen used in this case did not follow national or international guidelines and represented an individualized, off-label approach chosen for an exceptional and life-threatening situation. Although the patient recovered under this strategy, the regimen cannot be generally recommended, and its potential risks, including the development of drug resistance or disease recurrence, remain unknown.

## Data Availability

The original contributions presented in the study are included in the article. Further inquiries can be directed to the corresponding author.

## References

[1] Global tuberculosis report 2024 [https://www.who.int/publications/b/74877]

[2] Sharma SK, Mohan A. Miliary tuberculosis. Microbiol Spectr. 2017;5(2). 10.1128/microbiolspec.tnmi7-0013-2016 PMC11687475 28281441

[3] Sharma SK, Mohan A, Sharma A, Mitra DK. Miliary tuberculosis: new insights into an old disease. Lancet Infect Dis. 2005;5(7):415–30. 15978528 10.1016/S1473-3099(05)70163-8

[4] Kethireddy S, Light RB, Mirzanejad Y, Maki D, Arabi Y, Lapinsky S, Simon D, Kumar A, Parrillo JE, Kumar A. Mycobacterium tuberculosis septic shock. Chest. 2013;144(2):474–82. 23429859 10.1378/chest.12-1286

[5] Binh NG, Manabe T, Co DX, Thach PT, Tuan DQ, Cuong BV, Tuyet LTD, Kudo K, Anh NQ. Tuberculosis-induced acute respiratory distress syndrome treated with veno-venous extracorporeal membrane oxygenation. Respir Med Case Rep. 2019;28:100900. 31341764 10.1016/j.rmcr.2019.100900 PMC6630013

[6] Ait Hssain A, Petit M, Wiest C, Simon L, Al-Fares AA, Hany A, Garcia-Gomez DI, Besa S, Nseir S, Guervilly C, et al. Extracorporeal membrane oxygenation for tuberculosis-related acute respiratory distress syndrome: An international multicentre retrospective cohort study. Crit Care. 2024;28(1):332. 39385275 10.1186/s13054-024-05110-y PMC11465915

[7] Kethireddy S, Light RB, Mirzanejad Y, Maki D, Arabi Y, Lapinsky S, Simon D, Kumar A, Parrillo JE. Kumar A: <em>Mycobacterium tuberculosis</em> Septic Shock. Chest. 2013;144(2):474–82. 23429859 10.1378/chest.12-1286

[8] Dave SB, Leiendecker E, Creel-Bulos C, Miller CF, Boorman DW, Javidfar J, Attia T, Daneshmand M, Jabaley CS, Caridi-Schieble M. Outcomes following additional drainage during veno-venous extracorporeal membrane oxygenation: A single-center retrospective study. Perfusion. 2025;40(3):647–56. 38756070 10.1177/02676591241249609

[9] Hoofnagle JH, Björnsson ES. Drug-Induced Liver Injury - Types and Phenotypes. N Engl J Med. 2019;381(3):264–73. 31314970 10.1056/NEJMra1816149

[10] Sintusuwan M, Prueksapanich P, Suwanpimolkul G. Prevalence and risk factors associated with rifampicin-induced cholestasis jaundice among tuberculosis patients in high-incidence setting: A retrospective cohort study. J Clin Tuberc Other Mycobact Dis. 2026;43:100590. 41742903 10.1016/j.jctube.2026.100590 PMC12930029

[11] Alsultan A, Peloquin CA. Therapeutic drug monitoring in the treatment of tuberculosis: an update. Drugs. 2014;74(8):839–54. 24846578 10.1007/s40265-014-0222-8

[12] Idris R, Zielbauer AS, Koepsell J, Kloka J, Wetzstein N. Extracorporeal membrane oxygenation (ECMO) in patients with tuberculosis: systematic review and meta-analysis of 43 cases. BMC Pulm Med. 2024;24(1):47. 38254072 10.1186/s12890-023-02715-x PMC10801979

[13] Saukkonen JJ, Duarte R, Munsiff SS, Winston CA, Mammen MJ, Abubakar I, Acuña-Villaorduña C, Barry PM, Bastos ML, Carr W, et al. Updates on the Treatment of Drug-Susceptible and Drug-Resistant Tuberculosis: An Official ATS/CDC/ERS/IDSA Clinical Practice Guideline. Am J Respir Crit Care Med. 2025;211(1):15–33. 40693952 10.1164/rccm.202410-2096ST PMC11755361

[14] Pyrazinamide. LiverTox: Clinical and Research Information on Drug-Induced Liver Injury. edn. Bethesda (MD): National Institute of Diabetes and Digestive and Kidney Diseases; 2012.

[15] Rifampin. LiverTox: Clinical and Research Information on Drug-Induced Liver Injury. edn. Bethesda (MD): National Institute of Diabetes and Digestive and Kidney Diseases; 2012.

[16] Donald PR, Sirgel FA, Botha FJ, Seifart HI, Parkin DP, Vandenplas ML, Van de Wal BW, Maritz JS, Mitchison DA. The early bactericidal activity of isoniazid related to its dose size in pulmonary tuberculosis. Am J Respir Crit Care Med. 1997;156(3 Pt 1):895–900. 9310010 10.1164/ajrccm.156.3.9609132

[17] Fox GJ, Menzies D. A Review of the Evidence for Using Bedaquiline (TMC207) to Treat Multi-Drug Resistant Tuberculosis. Infect Dis Ther. 2013;2(2):123–44. 25134476 10.1007/s40121-013-0009-3 PMC4108107

[18] Mok J, Lee M, Kim DK, Kim JS, Jhun BW, Jo K-W, Jeon D, Lee T, Lee JY, Park JS, et al. 9 months of delamanid, linezolid, levofloxacin, and pyrazinamide versus conventional therapy for treatment of fluoroquinolone-sensitive multidrug-resistant tuberculosis (MDR-END): a multicentre, randomised, open-label phase 2/3 non-inferiority trial in South Korea. Lancet. 2022;400(10362):1522–30. 36522208 10.1016/S0140-6736(22)01883-9

[19] Hong H, Dooley KE, Starbird LE, Francis HW, Farley JE. Adverse outcome pathway for aminoglycoside ototoxicity in drug-resistant tuberculosis treatment. Arch Toxicol. 2019;93(5):1385–99. 30963202 10.1007/s00204-019-02407-8 PMC6667179

[20] Kühn D, Metz C, Seiler F, Wehrfritz H, Roth S, Alqudrah M, Becker A, Bracht H, Wagenpfeil S, Hoffmann M, et al. Antibiotic therapeutic drug monitoring in intensive care patients treated with different modalities of extracorporeal membrane oxygenation (ECMO) and renal replacement therapy: a prospective, observational single-center study. Crit Care. 2020;24(1):664. 33239110 10.1186/s13054-020-03397-1 PMC7689974

[21] Cendon L, Rafecas Codern A, de la Rosa D, Castellví I, Spagnolo P, Castillo D. Systematic Review of Systemic Corticosteroids for Treatment of Organizing Pneumonia. Open Respir Arch. 2022;4(4):100211. 37496960 10.1016/j.opresp.2022.100211 PMC10369534

[22] Wakamatsu K, Nagata N, Kumazoe H, Honjo S, Hamada M, Katsuki K, Hara M, Nagaoka A, Noda N, Kiyotani R, et al. Efficacy of steroid pulse therapy for miliary tuberculosis complicated by acute respiratory distress syndrome. J Clin Tuberc Other Mycobact Dis. 2022;29:100341. 36466135 10.1016/j.jctube.2022.100341 PMC9708912

